# Optimization to the Phellinus experimental environment based on classification forecasting method

**DOI:** 10.1371/journal.pone.0185444

**Published:** 2017-09-28

**Authors:** Zhongwei Li, Yuezhen Xin, Xuerong Cui, Xin Liu, Leiquan Wang, Weishan Zhang, Qinghua Lu, Hu Zhu

**Affiliations:** 1 College of Computer and Communication Engineering, China University of Petroleum, Qingdao 266580, Shandong, China; 2 College of Chemistry and Materials, Fujian Normal University, Fuzhou 350007, China; Xiamen University, CHINA

## Abstract

Phellinus is a kind of fungus and known as one of the elemental components in drugs to avoid cancer. With the purpose of finding optimized culture conditions for Phellinus production in the lab, plenty of experiments focusing on single factor were operated and large scale of experimental data was generated. In previous work, we used regression analysis and GA Gene-set based Genetic Algorithm (GA) to predict the production, but the data we used depended on experimental experience and only little part of the data was used. In this work we use the values of parameters involved in culture conditions, including inoculum size, PH value, initial liquid volume, temperature, seed age, fermentation time and rotation speed, to establish a high yield and a low yield classification model. Subsequently, a prediction model of BP neural network is established for high yield data set. GA is used to find the best culture conditions. The forecast accuracy rate more than 90% and the yield we got have a slight increase than the real yield.

## 1 Introduction

Phellinus is a kind of fungus having great medicinal value, since it is known as one of the elemental components in drugs avoiding cancers [1, 2]. Phellinus flavonoids is one of the most popular parasitifer of Phellinus in nature [3]. The research on Phellinus focuses on polysaccharides, proteoglycans medicinal mechanism, composition, etc., which are mostly extracted from the fruiting bodies of Phellinus flavonoids [4]. Phellinus rarely exists in the wild environment [5]. Cultivating Phellinus in the lab becomes a promising research branch. With mycelial growth by liquid fermentation, the fermentation broth flavonoids, polysaccharides, alkaloids and other active substances can be produced. These products have high level physical activity, short fermentation period and mass productions, thus providing a possible way of producing Phellinus in the lab [6]. In recent years, updated machine learning approaches [7, 8] have been developed and applied in biological data processing.

From the understanding of the wild conditions of Phellinus, it is found that PH value, temperature and fermentation time have an effect on the productions. As well, in general bio-chemical experiments, we need to consider the inoculum size, initial liquid volume, seed age and rotation speed [9, 10]. In the laboratory, plenty of experiments have been designed and operated for maximizing the Phellinus production.

Artificial algorithms and models have been used in the bio-process, particularly for the optimization of culture conditions. In [11], artificial neural networks (ANN) is used to optimize the extraction process of azalea Flavonoids. Neural networks combined with evolutionary algorithms have been used to optimize the experimental environment. For example, neural network and particle swarm optimization method is used for finding optimized culture conditions to maximize the Production of Pleuromutilin from Pleurotus Mutilus in [12]. The concept of classification is to learn a classification function on the basis of existing data or to construct a classification model (that is, what we usually call classifier). The function or model can map data records in the database to a given category. It can be applied to data prediction [13, 14]. Recently, many significant artificial intelligent algorithms and data processing strategies has been applied on data mining, such as a self-adaptive artificial bee colony algorithm based on global best for global optimization [15], the public auditing protocol with novel dynamic structure for cloud data [16], privacy-preserving smart semantic search method for conceptual graphs over encrypted outsourced data [17], a privacy-preserving and copy-deterrence content for image data processing with retrieval scheme in cloud computing [18] and machine learning method have been applied for experimental condition design, see. e.g. a secure and dynamic multi-keyword ranked search scheme over encrypted cloud data [19].

Genetic Algorithm (GA) derives from the computer simulation study of biological system [20], which has been widely used function optimization, combinatorial optimization, job shop scheduling problems [21], complex network clustering, pattern mining [22–24]. However, there are still some disadvantages, the most obvious disadvantages are the low efficiency and easy to fall into local optimum [25, 26].

In our previous paper in [27], we use the data collected during these experiments and take the statistical methods to establish a mathematical model in order to forecast the Flavonoid yield. Flavonoid yield is the most important product of Phellinus. With the purpose of finding the best Phellinus culture environment, the mathematical model was used as the fitness function for the GA and the result was developed. The result we got shows closely correspondence to the conclusion given by biologist. But during this process, the data we chosen to establish the mathematical model mainly rely on the prior knowledge of biologists. So we only use a little part of the whole data set. So we miss some information. Besides, the method does not work well in some areas where a priori knowledge lacked. In addition, the regression or BP neural network model established on all data sets can not get a accurate result. Therefore, in this paper, we use the classification algorithm for the whole sample set and achieve a good classification accuracy. On the basis of the high yield data set, the BP neural network and GA are used to optimize the yield. Finally, we find a better result than our previous work and the real data. This method can be used more extensively in biological experiments.

## 2 Data collected and data classification

### 2.1 Data collected

In this section, biological experiments are performed for finding optimal value of certain single factor.

In Table 1, experiments are operated for collecting data. In rows 1-14, it is associated with experiments with PH values ranging from 1 to 14, where the temperature is fixed to 28°C, Initial volume is set to be 100ml, the Rotation speed is 140r/m and seed age is 8 days. Rows 15 to 20 are 6 experiments with Initial volume ranges from 40ml to 140ml, where PH value is set to be 6, the best one obtained from experiments with PH values ranging from 1 to 14.

**Table 1:** Experiments with PH values ranging from 1 to 14 and initial volume ranges from 40ml to 140ml.

PH	Temp	Initial volume	Rotation speed	Including inoculum	seed age	Fermentation time	Phellinus yield (*μ*g/ml)	class
1	28°C	100ml	140	5%	8	8	45.929	0
2	28°C	100ml	140	5%	8	8	35.077	0
3	28°C	100ml	140	5%	8	8	45.654	0
4	28°C	100ml	140	5%	8	8	534.39	0
5	28°C	100ml	140	5%	8	8	702.81	0
6	28°C	100ml	140	5%	8	8	1467.7	1
7	28°C	100ml	140	5%	8	8	189.20	0
8	28°C	100ml	140	5%	8	8	91.049	0
9	28°C	100ml	140	5%	8	8	60.841	0
10	28°C	100ml	140	5%	8	8	57.225	0
11	28°C	100ml	140	5%	8	8	43.238	0
12	28°C	100ml	140	5%	8	8	36.288	0
13	28°C	100ml	140	5%	8	8	20.943	0
14	28°C	100ml	140	5%	8	8	22.306	0
6	28°C	40ml	140	5%	8	8	508.495	0
6	28°C	60ml	140	5%	8	8	900.662	0
6	28°C	80ml	140	5%	8	8	1273.594	1
6	28°C	100ml	140	5%	8	8	1153.937	0
6	28°C	120ml	140	5%	8	8	1123.330	0
6	28°C	140ml	140	5%	8	8	1088.064	0

In Table 2, experiments with Including inoculum ranging from 2% to 16% and Temperature ranging from 25°C to 40°C are performed. That the situations on experiments with Fermentation time ranging from 1 to 12 hours are shown in Table 3. From the total 45 experiments, we collect data of culture conditions for production of Phellinus. Different culture conditions have a fundamental influence on the production of Phellinus. However, the optimized culture conditions remain unknown.

**Table 2:** Experiments with including inoculum ranging from 2% to 16% and temperature ranging from 25°C to 40°C.

PH	Temp	Initial volume	Rotation speed	Including inoculum	seed age	Fermentation time	Phellinus yield (*μ*g/ml)	class
6	28°C	100ml	140	2%	8	8	546.609	0
6	28°C	100ml	140	4%	8	8	606.345	0
6	28°C	100ml	140	6%	8	8	1320.794	1
6	28°C	100ml	140	8%	8	8	1447.519	1
6	28°C	100ml	140	10%	8	8	1841.729	1
6	28°C	100ml	140	12%	8	8	1631.990	1
6	28°C	100ml	140	14%	8	8	481.1172	0
6	28°C	100ml	140	16%	8	8	449.5187	0
6	25°C	40ml	140	10%	8	8	1145.669	0
6	30°C	60ml	140	10%	8	8	1506.055	1
6	35°C	80ml	140	10%	8	8	1374.982	1
6	40°C	100ml	140	10%	8	8	875.341	0

**Table 3:** Experiments with fermentation time ranging from 1 to 12 hours.

PH	Temp	Initial volume	Rotation speed	Including inoculum	seed age	Fermentation time	Phellinus yield (*μ*g/ml)	class
6	28°C	100ml	150	2%	8	1	56.606	0
6	28°C	100ml	150	4%	8	2	83.435	0
6	28°C	100ml	150	6%	8	3	303.984	0
6	28°C	100ml	150	8%	8	4	449.919	0
6	28°C	100ml	150	10%	8	5	777.331	0
6	28°C	100ml	150	12%	8	6	1103.987	0
6	28°C	100ml	150	14%	8	7	1619.554	1
6	28°C	100ml	150	16%	8	8	1597.995	1
6	28°C	100ml	150	10%	8	9	1546.336	1
6	28°C	100ml	150	10%	8	10	1502.487	1
6	28°C	100ml	150	10%	8	11	1489.364	1
6	28°C	100ml	150	10%	8	12	1465.664	1

### 2.2 Data classification

In this section, we consider to divide the data set into high yield data set and low yield data set two parts. In our previous work, we found that the data collected from biological experiment has similarity and the gradient is limited. The conventional prediction method is difficult to achieve good results in the whole data set. So we use the method of classification, only focus on some important data, and increase the sample difference in the classified data set. There are two factors that must be considered. The fist one, we need to keep the balance between two data sets [28]. Larger imbalances can lead to more deviations in our classifiers. For example, we have one set of high yield data and 99 sets of low yield data, it is clear that the prediction of low yield data can reach 99% without learning, but the classifiers may not reach 99%. This is the imbalance caused by the data. Even the accuracy of the model is high, the model is certainly not good in the prediction of high yield data and not the model we want. If we use this model, our classifier can not find the high yield factors and provide a training data set for BP neural network to establish a prediction model. The second one, the high yield data set and low yield data set must cover all single factor experimental conditions.

Now we have two classification strategies. The first one, we take the median of flavonoid production as the classification boundary (in our experiment is 1100*μ*g/ml) and we have the same number of high-yield collections and low-yield collections. We have done a number of experiments to prove that the classification effect is acceptable. We can see the classification results in Table 4. But we realized that this classification method will lead to a single factor test of a class completely classified as high yield or low production set. In our experiment, all data belong to the seed age factor will be divided into high yield data set. Seed age for our classifier is no longer a decision-making factor which will lead to a large prediction error. We can see it in Table 5.

**Table 4:** 1100*μ*g/ml boundary classification accuracy (logical regression).

Type	0	1	The correct percentage
0	20	6	76.9
1	3	11	88
total			82.4

**Table 5:** Experiments with seed age ranging from 4 to 10 hours.

PH	Temp	Initial volume	Rotation speed	Including inoculum	seed age	Fermentation time	Phellinus yield (*μ*g/ml)	class
6	28°C	100ml	150	2%	4	1	1272.384	0
6	28°C	100ml	150	4%	5	2	1453.231	1
6	28°C	100ml	150	6%	6	3	1428.025	1
6	28°C	100ml	150	8%	7	4	1477.273	1
6	28°C	100ml	150	10%	8	5	2164.513	1
6	28°C	100ml	150	12%	9	6	2127.726	1
6	28°C	100ml	150	14%	10	7	1741.498	1

Another strategy is to select a boundary in each set of univariate experimental data to keep the data for each single factor experiment in two different classes, while keeping the number of elements in the two categories as close as possible. In combination with the above conditions, we chose the flavonoid yield equal to 1273 *μ*g/ml as our boundary condition. Under this boundary condition, we obtain 20 sets of high yield data and 30 sets low yield data, which include the conditions of each group of single factor experiments. We can see the classification results in Table 6.

**Table 6:** 1273*μ*g/ml boundary classification accuracy (logical regression).

Type	0	1	The correct percentage
0	21	10	67.7
1	4	16	80
total			72.5

## 3 Methods

Our experiment is mainly composed of three parts. The first part, the high-yielding data set is determined by the classification model, and then BP neural network is used to forecast. Finally, the parameters of BP neural network and the threshold are used as fitness function to find the optimal yield with GA.

### 3.1 Classification model

From the above boundary we determine the high yield and low yield of two data sets, the high yield is set to be 1 and the low yield is set to be 0. We use two classifiers to identify the classification effect, logical regression and BP neural network classifier. we use the SMOTE algorithm to improve the data set [29]. The idea of the SMOTE algorithm is to synthesize new samples of minority class (the high yield class). The synthetic strategy is to choose A’s nearest neighbor B for each sample of minority class, and then random select a new sample as a minority class sample between A and B [30]. This hybrid computational method, which combines with SVM and AGA, has the intelligent learning ability and can overcome the limitation of large-scale biotic experiments [31–36].

(1) for each sample X in a minority classes, the distance of all samples is computed from the Euclidean distance as the criterion, and the k nearest neighbor is obtained.

(2) according to the sample imbalance ratio, a sampling ratio is set to determine the sampling rate N. For each minority class sample x, several samples are selected randomly from their K neighbors, assuming that the nearest neighbor is xn.

(3) for each randomly selected neighbor xn, a new sample is constructed according to the following formula *xm* = *x* + *rand*(0,1) * (*xn* − *x*). The xm is the new sample.

Compared with other data expansion methods, SMOTE algorithm generates new data instead of directly copying minority class samples. This can increase sample differences within class. We know that biological experiments set up certain experimental gradients to carry out a set of experiments. And the variation of adjacent experimental gradient data is usually linear. For example, if the PH value is 5, and corresponding yield is 300, the PH is 6, and corresponding yield is 1000, the PH is 7, and corresponding yield is 500. We usually think that when PH is 5.5, the yield is between 300 and 1000. If we set the classification boundaries yield is 300, then PH is 5.5 and can be divided into a few samples. In this way, we increase the sensitivity of the classifier to some experimental conditions and improve the accuracy of classification. We don’t use these new generated samples for production forecasting because we are not sure of their exact yields.

In each of our experiments, each experiment gradient was set as a unit to compare the distance between each experiment. Since the number of samples we divide into two categories is different, there is no doubt that classification results are better for most sets. In addition, the overall number of samples is small and the classification effect fluctuates greatly. SMOTE algorithm is used to increase the sample size of the minority class, which is more balanced in the overall distribution of the data, while increasing the number of samples as a whole, reducing volatility. We can see that the classification effect has been improved by SMOTE algorithm in Tables 7 and 8.

**Table 7:** 1273*μ*g/ml boundary classification accuracy after SMOTE (logical regression).

Type	0	1	The correct percentage
0	21	10	67.7
1	3	27	90
total			79.7

**Table 8:** Comparison of the effects of SMOTE algorithm processing and data processing without SMOTE algorithm.

Type	without SMOTE	with SMOTE
logical regression	72.5	79.7
BP	80	87

The correct percentage = *z*;

The predicted yield = *y*;

The active yield = *x*;

*z* = |(*y*−*x*)/*x*|;

In this section, we establish a reliable classification model that can classify high yield and low yield data and then predict the yield in the next step if the experimental conditions belong to high yield data set.

### 3.2 BP neural network

BP (Back Propagation) neural network was developed by Rumelhart and McClelland in 1986. BP is a multi-layer feed forward neural network trained by error back propagation algorithm and it is the most widely used neural network [37].

The basic BP algorithm includes the forward propagation of the signal and the reverse propagation of the error. We calculate the error output from the input to the output direction, and adjust the weight and threshold from the output to the input direction. After training, the trained neural network that can be similar to the sample input information, the minimum output error is used to deal with the non-linear conversion of information [38, 39].

**Fig 1:**
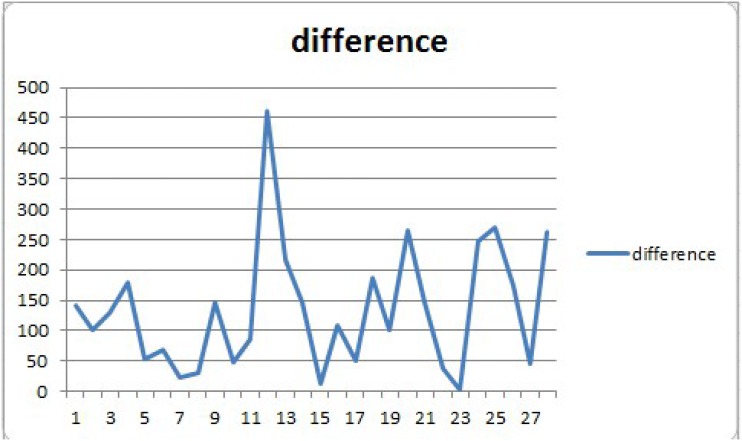
The difference between the real value and the predicted value.

**Fig 2:**
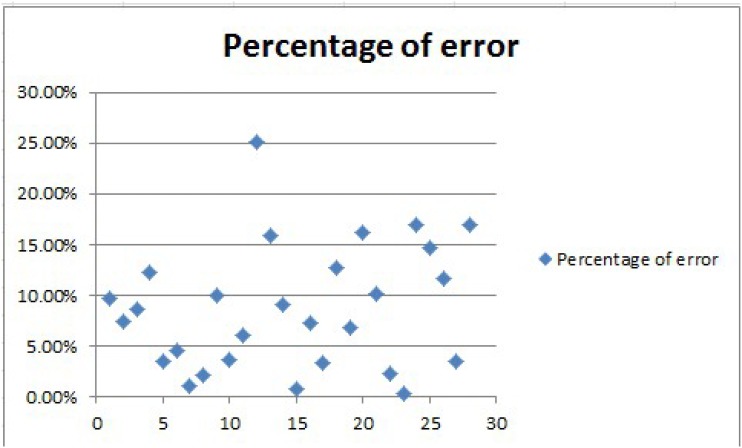
Percentage of error.

Each time we randomly selected 16 sets of data as a training set, the establishment of a experimental conditions and output corresponding to the forecast model. 4 sets of data as a test set, used to verify the reliability of modeling. Repeat seven experiments. We can see the result in Table 9. After repeated tests, the number of intermediate layer nodes is determine to be 9. Each hidden layer transfer function is set to be “tansig”, “logsig”, “tansig”. The training function is set to be “trainlm”. Each time 15 sets of data are selected for modeling. Five sets of data are selected to verify. Times of training is set to be 1000, training convergence error is set to be 0.00001. The results of repeat seven experiments as follows. The average error is 133.53, the percentage of error is 8.7%. The error value is shown in Fig 1 and percentage of error is shown in Fig 2. We can judge that our model has achieved a good result.

**Table 9:** Experimental results.

Type	Actual yield	Forecast yield	error	Percentage of error
1	1447.519173	1587.9	140.3808272	9.7%
2	1374.982592	1273.6	101.382592	7.3%
3	1502.487	1632	129.513	8.62%
4	1453.230569	1274.9	178.3305688	12.27%
5	1506.05569	1453.0896	52.9660896	3.52%
6	1489.364	1420.734	68.63	4.61%
7	2127.725793	2103.7928	23.9329928	1.12%
8	1453.230569	1423.2688	29.9617688	2.06%
9	1467.790541	1321.5	146.2905408	9.97%
10	1273.594991	1320.8	47.2050088	3.71%
11	1447.519173	1360.8	86.7191728	5.99%
12	1841.729358	1380.6	461.1293584	25.04%
13	1374.982592	1592.9	217.917408	15.85%
14	1619.554	1473.6	145.954	9.01%
15	1597.995	1586.4	11.595	0.73%
16	1502.487	1394.3	108.187	7.20%
17	1506.05569	1454.8	51.2556896	3.40%
18	1465.664	1278.7	186.964	12.76%
19	1477.273482	1376.9	100.3734816	6.79%
20	1631.990382	1368.2	263.7903824	16.16%
21	1447.519173	1300.50	147.0191728	10.16%
22	1597.995	1560.90	37.095	2.32%
23	1320.794994	1317.00	3.7949936	0.29%
24	1453.230569	1699.80	246.5694312	16.97%
25	1841.729358	1571.40	270.3293584	14.86%
26	1489.364	1315.70	173.664	11.66%
27	1320.794994	1274.00	46.7949936	3.54%
28	1546.336	1285.30	261.036	16.88%

The Forecast yield is the yield calculated by the BP neural network under the same experimental conditions.

The actual yield = *x*;

The Forecast yield = *y*;

error = *z*

*z* = |*x*−*y*|

The percentage of error = *z*/*x*

In this section, we build a prediction model for high yield data sets and verify its reliability.

### 3.3 GA process

In this part we use the established model and GA to optimize the yield.

**Fig 3:**
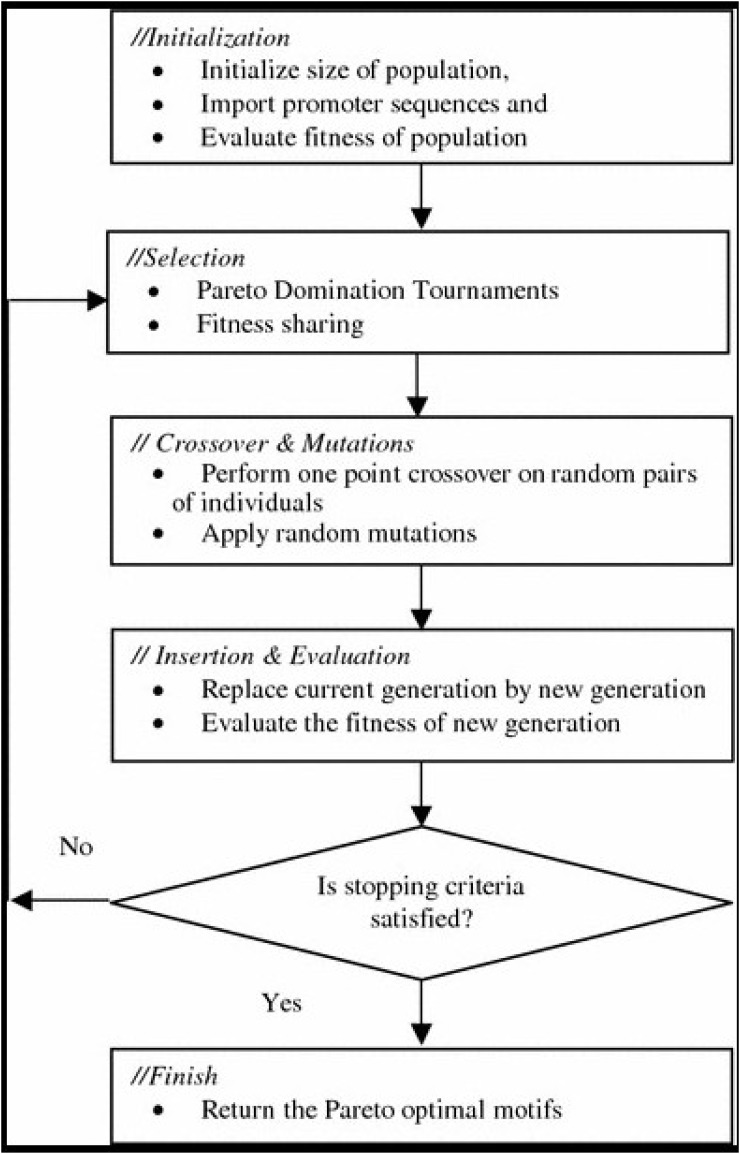
GA process.

Genetic algorithm is a kind of randomized search method which is based on the evolution of biological circles [40]. It was first proposed by Professor J. Holland of the United States in 1975 [41]. Its main feature is that it directly operates on structural objects without the existence of derivative and function continuity; with inherent implicit parallelism and better global optimization. GA use probabilistic optimization method, it can automatically obtain and guide the optimization of the search space [42]. These properties of genetic algorithms have been widely used in the fields of combinatorial optimization, machine learning, signal processing, adaptive control and artificial life. It is the modern key technology in intelligent computing [43]. The GA process is in Fig 3.

**Fig 4:**
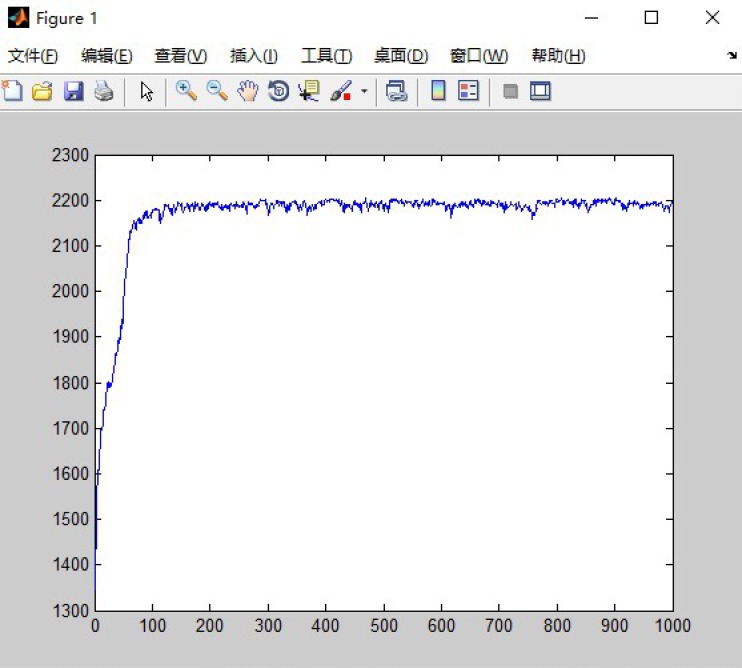
GA result after training.

The parameters for setting the GA algorithm are as follows: population size is set to be 300, chromosome size is set to be 6, generation size is set to be 1000, cross rate is set to be 1, mutate rate is set to be 0.01. The mutation rate and cross rate affect the number of iterations and iterations of the GA process. Because the number of iterations we set is much more than the actual number of iterations required. So after many tests, the mutation rate is set to be minimum value and cross rate is set to be maximum value. This is the ideal condition of the genetic algorithm. The encoding mechanism is real-number encoding. The hidden threshold of BP neural network is extracted as the fitness function of GA algorithm. After about 30 to 500 iterations the GA process returns the best individual. The training process is in Fig 4. Repeat the test seven times and result as follow in Table 10. We can see that the yield we got have a slight increase than the real yield.

**Table 10:** Optimal conditions and yield obtained by simulation.

PH	Temp	Initial volume	Rotation speed	Including inoculum	seed age	Fermentation time	Phellinus yield (*μ*g/ml)	Iterations
6	29°C	100ml	150	12%	7	8	2164.8	39
6	28°C	90ml	150	12%	8	11	2204.1	31
6	30°C	90ml	150	12%	7	12	2121.6	208
6	30°C	90ml	141	9%	8	8	2045.2	430
6	28°C	90ml	150	12%	8	11	2204.1	52
6	29°C	100ml	150	12%	9	11	2207.6	44
6	29°C	100ml	150	12%	8	8	2171.8	56

In this section, we use the weight threshold of BP neural network as the optimization object, and use the GA algorithm to find the optimal experimental conditions.

## 4 Conclusion

In this work, we firstly classify the collected data sets and establish a classification model. Classification accuracy rate can reach more than 80%. We use our selected high-yielding data set for modeling. Forecast accuracy rate more than 90%. Finally, the weight threshold of BP neural network is used as the fitness function of GA to optimize the yield. So we have established a set of mulberry flavonoids production forecast and optimization process. When the biologist give us a new set of experimental conditions, we first use the classification model to verify whether these conditions are high-yield conditions. If these conditions are high-yield conditions, we use the established BP neural network to predict the yield. In the comparison results, it is believed that PH value is credible 6 and the temperature is also within the appropriate temperature range 28°C to 30°C. Taking into account environmental factors in the laboratory, the initial volume, rotation speed and including inoculum we predicted are also reliable. The seed age is 7 or 8 closing to the original data 8. The fermentation time predicted rang from 8 to 11 more than the original data 8. However, iit can be explained in terms of biological experiments. When the fermentation time reaches a certain limit after the mulberry community to reach the limit, this time the output depends mainly on the supply of nutrients, so the data we get is acceptable. The average Phellinus yield we predicted is 2159.9*μ*g/ml more than the original data 2127*μ*g/ml. Data experimental results show that predicted optimal values of the parameters have accordance with biological experimental results, which indicate that our method has a good predictability for culture conditions optimization.

For further research, neural-like computing models, e.g., spiking neural P systems [44] can be used for optimization of Welan gum production. As well, some recently developed data processing and mining methods, such as the speculative approach to spatial-temporal efficiency for multi-objective optimization in cloud data and computing [45], privacy-preserving smart similarity search methods in simhash over encrypted data in cloud computing [45], k-degree anonymity with vertex and edge modification algorithm [46], kernel quaternion principal component analysis for object recognition [47], might be used for Optimization to the Phellinus Experimental Environment. In the aspect of data preparation, decision tree [48] can be used to deal with the missing attribute value of some samples in dataset.
